# Metagenomic Analysis of Airborne Bacterial Community and Diversity in Seoul, Korea, during December 2014, Asian Dust Event

**DOI:** 10.1371/journal.pone.0170693

**Published:** 2017-01-25

**Authors:** Seho Cha, Sathiyaraj Srinivasan, Jun Hyeong Jang, Dongwook Lee, Sora Lim, Kyung Sang Kim, Weonhwa Jheong, Dong-Won Lee, Eung-Roh Park, Hyun-Mi Chung, Joonho Choe, Myung Kyum Kim, Taegun Seo

**Affiliations:** 1 Department of Life Science, Dongguk University-Seoul, Goyang, South Korea; 2 Department of Bio & Environmental Technology, Division of Environmental & Life Science, College of Natural Science, Seoul Women’s University, Seoul, South Korea; 3 Biosafety Research Team, Environmental Health Research Department, National Institute of Environmental Research, Incheon, South Korea; 4 Air Quality Research Division, Climate and Air Quality Research Department, National Institute of Environmental Research, Incheon, South Korea; 5 Water Supply and Sewerage Research Division, Environmental Infrastructure Research Department, National Institute of Environmental Research, Incheon, South Korea; 6 Department of Biological Sciences, Korea Advanced Institute of Science and Technology, Daejeon, South Korea; AC Camargo Cancer Hospital, BRAZIL

## Abstract

Asian dust or yellow sand events in East Asia are a major issue of environmental contamination and human health, causing increasing concern. A high amount of dust particles, especially called as particulate matter 10 (PM10), is transported by the wind from the arid and semi-arid tracks to the Korean peninsula, bringing a bacterial population that alters the terrestrial and atmospheric microbial communities. In this study, we aimed to explore the bacterial populations of Asian dust samples collected during November–December 2014. The dust samples were collected using the impinger method, and the hypervariable regions of the 16S rRNA gene were amplified using PCR followed by pyrosequencing. Analysis of the sequencing data were performed using Mothur software. The data showed that the number of operational taxonomic units and diversity index during Asian dust events were higher than those during non-Asian dust events. At the phylum level, the proportions of Proteobacteria, Actinobacteria, and Firmicutes were different between Asian dust and non-Asian dust samples. At the genus level, the proportions of the genus *Bacillus* (6.9%), *Arthrobacter* (3.6%), *Blastocatella* (2%), *Planomicrobium* (1.4%) were increased during Asian dust compared to those in non-Asian dust samples. This study showed that the significant relationship between bacterial populations of Asian dust samples and non-Asian dust samples in Korea, which could significantly affect the microbial population in the environment.

## Introduction

Asian dust events in East Asia, including Korea and Japan, are a seasonal phenomenon, mostly in the early spring that influences the airborne environment and human health problems [1–3]. Asian dust is known to originate from several arid regions of China, including the Gobi desert and the Taklamakan desert, and the dust particles affect the surrounding countries, including China, Japan, and Korea [4–6], as well as Greenland and North America through the global transport of Asian dust particles [7–9]. Moreover, Asian dust events have increased from 77 days containing 8 days during winter (Dovember to February) in 1990s to 122 days containing 21 days during winter in 2000s (Korea meteorological administrate, KMA).

During Asian dust events, several hundred times the normal level of dust particles are present in the airborne environment, and the dust particles contain various chemical components as well as microorganisms [10–13]. Previous studies have shown that an increasing concentration of the dust particles is associated with various human health problems and several chemical components or heavy metals found in the dust particles are known to contribute various human diseases [14, 15]. However, a report by Kim *et al*. showed that the airborne Hg concentration was not different during Asian dust events [16], and He *et al*. showed that heat-treated Asian dust samples did not induce allergic lung inflammation compared to normal dust samples, suggesting that unknown microbial materials could contribute some risk to human health [17]. In recent years, the chemical compositions of Asian dust particles have been identified comparatively, but the microbial community structure of the dust particles is not yet understood. Only limited studies have reported that the bacterial communities in an airborne environment were altered during Asian dust events, and exposure to some of the microorganisms could cause some human health problems [18–20]. These studies have provided a biological feature of Asian dust particles, and are useful researches to investigate environmental effects of Asian dust events. However, since an Asian dust event is a seasonal phenomenon and bacterial communities in Asian dust particles are influenced by a variety of conditions, including origin region, sampling method, sampling method, and period, an accumulation of bacterial information through a continuous monitoring is important to understand an alteration of bacterial community structure during Asian dust events.

In this study, we aimed to investigate an alteration of bacterial communities during Asian dust and non-Asian dust events in December 2014, South Korea, and focused to determine a detailed taxonomic feature. For the characterization of airborne bacterial structure, we performed a metagenomics analysis using the next-generation sequencing method to overcome the limitations of culture-based techniques, which can only analyze culturable bacteria in a specific kind of medium. We analyzed bacterial communities and diversity features from the Asian dust and non-Asian dust events, and statistically investigated different phyla and genera between the two events. These results will be used to understand the alteration of bacterial communities by transported dust particles during Asian dust events and to provide information on airborne bacteria during the period November to December, 2014 in South Korea.

## Materials and Methods

### Collection of air samples

Asian dust and non-Asian dust event samples were collected on the rooftop of Dongguk University in Seoul, South Korea (GPS; 37.557 N, 127.002 E). No other commercial buildings surround the sampling building and it is free of the influence of other pollutant contamination. A liquid impinger connected with an aspirator was used to collect the dust samples on the rooftop (25 m height) of the building. The dust samples were collected in 300 ml of phosphate-buffered saline (PBS) for 10 to 24 hours (flow rate is 20 L/min). Asian dust event samples were collected from December 1 to December 2, 2014 and the non-Asian dust event samples were collected on November 26 and December 3, 2014 for comparison. The water-insoluble bioaerosols were filtered using a polycarbonate filter membrane and used for the analysis [21].

### Environmental information and backward trajectories of air during the sampling period

Meteorological information, including humidity, temperature, and particulate matter 10 (PM10) concentration, was obtained from the Korea meteorological administration (KMA). For the backward trajectory analysis of transported dust particles, the hybrid single-particle Lagrangian integrated trajectory (HYSPLIT) model was used [22, 23]. Global data assimilation system (GDAS) data provided by national centers for environmental prediction (NCEP) were used as meteorological prediction information for the trajectory analysis. The starting point of the backward trajectory analysis was the sampling location (GPS; 37.557 N, 127.002 E), and the starting time was 15 UTC (00 Korea standard time). To track the source of dust particles during the Asian dust and non-Asian dust events, 48 h backward trajectories were calculated at the height of 500 m.

### DNA isolation

After filtering the collected bioaerosol through a polycarbonate filter membrane, half of the membrane was used to extract genomic DNA. The genomic DNA was extracted by a previously described method [24]. In brief, half of the membrane was mixed with DNA extraction buffer (100 mM Tris-Cl, pH 8.0; 100 mM sodium-EDTA, pH 8.0; 100 mM sodium phosphate, pH 8.0; 1.5 M NaCl; 1% CTAB] and 1 mg of proteinase K, followed by incubation with shaking at 37°C for 30 min. Then, the mixture was incubated with 2% SDS at 65°C for 30 min. After centrifugation, the supernatants were mixed with an equal volume of chloroform:isoamylalcohol (24:1, v/v). The aqueous layer was mixed with 70% isopropanol and the precipitated DNA pellet was washed with 70% ethanol. The genomic DNA pellet was dried and resuspended with distilled water.

### Pyrosequencing and analysis

For amplification of the 16S rRNA gene region from the extracted DNA, a set of forward and reverse primers was constructed according to a previous report [25]. The forward primer sequence is 517F of the 16S rRNA gene region, GCCAGCAGCCGCGGTAAT, and the reverse primer sequence is 896R of the 16S rRNA gene region, CCGTACTCCCCAGGCGG. The region contains the fourth and fifth hypervariable regions of 16S rRNA gene sequences. For pyrosequencing, proper adaptor and multiplex identifier tag sequences provided by the manufacturer’s instructions (Macrogen, Korea) were conjugated with primer sequences. PCR was performed by following conditions: pre-denaturation at 95°C for 3 min, 35 cycles of denaturation at 95°C for 3 s, and annealing and extension at 64°C for 15 s. After amplification of the 16S rRNA gene region, equal amounts of each PCR product (30 ng) were collected in a new tube and pyrosequencing processes were performed by a GS FLX system (Roche, Switzerland). The sequences were submitted into the INSDC SRA (S1 File, BioProject and SRA accession numbers are PRJNA360153 and SRP096119, respectively). For analysis of the pyrosequencing results, we performed a standard procedure using Mothur software [26]. Briefly, trim.seq command was used to remove short and low quality sequences. Since the length of amplified PCR product had more than 300 bp, a threshold length of sequence was determined as more than 200 bp. A threshold of quality score value was determined by a default value in Mothur software (qwindowaverage value in trim.seq command is 35). After trimming the unnecessary sequences, chimera.uchime command was used to remove potentially chimeric sequences. The total read numbers for November 26 and December 1 to December 3 were 7269, 7119, 7873, and 8483, respectively. Bacterial classification and statistical analysis were performed using the SILVA database (release 119) [27] and Metastats software, respectively [28]. For calculating diversity indices, including OTU [29], Chao [30], and inversed simpson index [31], a taxonomic data-based phylotype analysis at species level was performed using Mothur software. Briefly, the bacterial classification data (taxonomy files in Mothur software) analyzed by the SILVA database were used to process phylotype command. We used a phylotype analysis instead of cutoff-based clustering, because several sequences were classified into a same taxonomic name even though the sequences had a different OTU at cutoff value of 0.03. In the present study, since we focused the respective taxonomic information for identifying a taxonomic characteristic between Asian dust and non-Asian dust events, the taxonomic data-based phylotype analysis was used. After producing shared files at species level using phylotype command, the files were used to analyze diversity indices, including OTU, Chao, and inversed simpson index. Bacterial proportion results and statistical data were analyzed using shared files at phylum and genus level.

## Results and Discussion

### Environmental conditions during the sampling period

**Fig 1:**
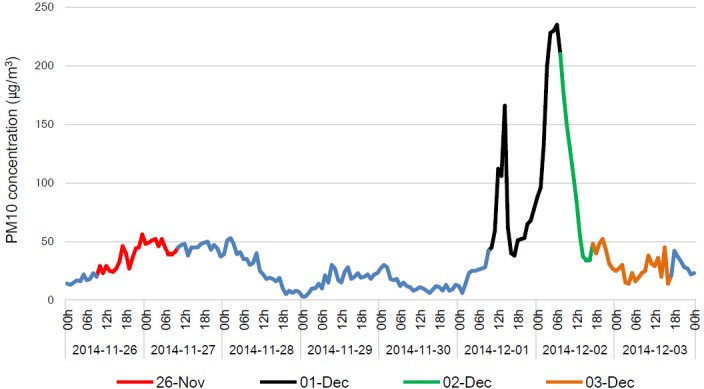
Concentration of PM10 during Asian dust and non-Asian dust events. Indicated date presents sampling time and the time is Korea standard time, KST (UTC + 09).

In order to determine any alterations to the bacterial community during Asian dust events, we collected airborne samples on November 26 and from December 1 to December 3. The relative humidity during the sampling period varied randomly around 57.6–79.3% and the temperature was about -2.7–6°C. No precipitation occurred during sampling period. The backward trajectory analysis showed that the air mass on November 26, 2014 originated from the East Sea. Meanwhile the air mass during December 1 to December 3 came from the desert area of Mongolia, passed over China, and arrived at the Korean peninsula for 2 days (HYSPLIT, data not shown). As shown in Fig 1, the PM10 concentration increased from December 1, and was more than 200 μg/m^3^ at 03:00–07:00 (Korea standard time, KST, UTC + 09) on December 2. Based on the hourly averaged PM10 concentration obtained from KMA and backward trajectory analysis, the collected airborne samples were classified into two group, Asian dust (December 1 and December 2) and non-Asian dust (November 26 and December 3). In this study, there are some different sampling durations between December 2 and other days. We, however, cautiously suggests that the different duration may not affect the following results since there are a similar biological characteristic between December 1 and 2. In addition, we performed SourceTracker software to confirm whether the samples were contaminated by other sources during sampling or following processes. Human microbiome project (HMP) data was used to compare a proportion of each sample under different source condition. In case of non-Asian dust as source, Asian dust samples had a relative high proportions, 0.8081 (December 1) and 0.7419 (December 2), but HMP data revealed clearly low proportion (0.1048). Moreover, the proportions of non-Asian dust samples (0.9007 in November 26) and 0.9564 in December 3) are clearly higher than HMP data (0.1876) in case of Asian dust as source.

### Diversity analysis of the bacterial communities in the Asian dust and non-Asian dust event samples

**Fig 2:**
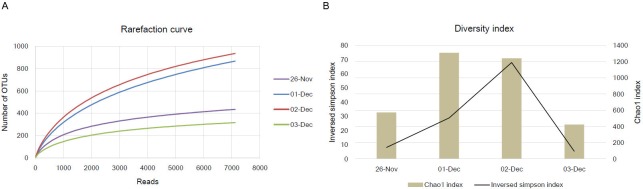
Rarefaction curves and diversity index of the Asian dust and non-Asian dust event samples.

After pyrosequencing of amplified 16S rRNA gene products obtained from November 26 and December 1 to December 3, we analyzed the sequences using the standard operating procedure of Mothur software. For normalization of each sample, we randomly selected 7119 sequences presented the lowest sequences among each sample. To analyze an alteration of the diversity index caused by newly transported dust particles containing variable microorganisms during the Asian dust events, we first determined operational taxonomic units (OTUs) of each sample at the species level. As shown in Fig 2A, the number of OTUs in the Asian dust sample were higher than those in the non-Asian dust sample (t-test, P = 0.02). In addition, the Chao1 index and the inversed Simpson index, which present a diversity index, were also increased in the Asian dust event samples (Fig 2B). Each diversity index was 2.55 times (t-test, P = 0.044) and 7.22 times (t-test, P = 0.13) higher than that for the non-Asian dust event samples. In a previous study, Jeon *et al*. also show that the diversity index, Chao1, of Asian dust event samples was 2.63 times higher than non-Asian dust event samples [32]. Indeed, while we expected that both richness (OTUs and Chao1) and evenness (Simpson) indices had a significantly difference value between bacterial communities of Asian dust and non-Asian dust, only richness indices were significantly higher in the Asian dust samples. However, we observed a consistent result in the other period (in submission), suggesting that while various kind of microorganisms were transported by Asian dust particles, several taxonomic groups were presented as a relatively higher proportion in the dust particles. Beside the concerned issue, these results mean that newly transported microorganisms are present in the airborne environment during Asian dust events, leading to an alteration of the airborne microbial community compared to its normal status.

### Characterization of bacterial community at the phylum level

**Fig 3:**
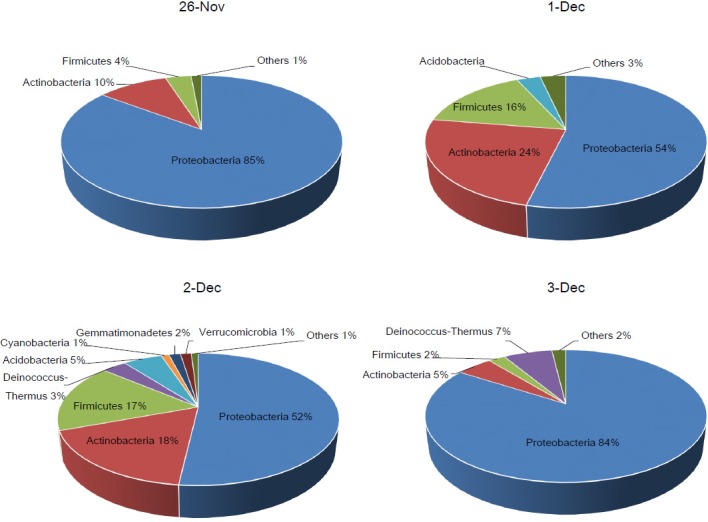
Bacterial communities (%) at the phylum level in the Asian dust and non-Asian dust event samples. Other phyla present lower than 1% of total bacteria community.

Through the diversity analysis, we confirmed that the airborne microbial environment was affected by transported dust particles, which probably contain various microorganisms, during Asian dust events. We therefore classified the sequences obtained from pyrosequencing data according to the SILVA database. As a result, a total of 19 phyla were classified and several predominant phyla (more than 1% of total bacterial community) are presented in Fig 3 and Table 1. From the results, we confirmed that the proportion of phylum Proteobacteria in the non-Asian dust samples was 1.6 times higher than the Asian dust samples. Meanwhile, the proportions of various phyla, including Actinobacteria, Acidobacteria, Firmicutes, and Gemmatimonadetes, were increased compared to those in the non-Asian dust event samples. To determine which phyla have a statistical difference between the two groups, we performed Metastats analysis using Mothur software. As shown in Table 2, the proportions of phyla Proteobacteria, Actinobacteria, and Firmicutes were significantly different between the two groups. In the Asian dust event samples, the phylum Proteobacteria was 0.62 times lower than in the non-Asian dust samples, and phyla Actinobacteria and Firmicutes were increased by 2.8 times and 5.6 times, respectively. These results mean that the proportion of the predominantly presented phylum, Proteobacteria, in the airborne environment was reduced with an increasing influx of newly transported microorganisms during Asian dust events. The influx of phyla Actinobacteria and Firmicutes during Asian dust events was also reported in previous work [32, 33]. As similar with this results, the phylum Proteobacteria was predominantly presented during non-Asian dust events (53.1%), while Firmicutes presented as a relatively low proportion. The proportions of phyla Proteobacteria and Firmicues were changed into 15% and 53%, but the proportion of Actinobacteria was rarely altered during Asian dust events [32]. In addition, Yamaguchi *et al*. showed that the phylum Actinobacteria was largely present in the Asian dust event samples as well as in several source soils of Asian dust, including the Gobi desert and the Taklamakan desert [34]. Although there are several difference between these studies, Proteobacteria is one of predominant phylum during normal days, and proportions of Firmicutes and Actinobacteria were mainly altered during Asian dust events. However, since environmental conditions, sampling methods, and regions are different in each research, it is important to accumulate various database for understanding biological features of Asian dust particles.

**Table 1:** Bacterial communities (%) at the phylum level in the Asian dust and non-Asian dust event samples.

Phylum	Bacterial communities (%)			
	26-Nov (non-Asian dust)	1-Dec (Asian dust)	2-Dec (Asian dust)	3-Dec (non-Asian dust)
**Proteobacteria**	85.29371	53.7716	51.83539	84.13297
**Actinobacteria**	9.739992	23.75334	17.50286	4.962867
**Firmicutes**	3.549319	15.69041	16.84237	2.298715
**Deinococcus-Thermus**	Tr	Tr^a^	3.391337	6.695744
**Acidobacteria**	Tr	3.258885	5.334688	Tr
**Cyanobacteria**	Tr	Tr	1.079639	Tr
**Gemmatimonadetes**	Tr	Tr	1.587705	Tr
**Verrucomicrobia**	Tr	Tr	1.486092	Tr
**Others**	1.416976	3.525776	0.939921	1.909702

^a^ Tr, trace, indicates a proportion lower than 1%.

**Table 2:** Metastats analysis at the phylum level in the Asian dust and non-Asian dust event samples.

Phylum	1-Dec and 2-Dec (Asian dust)			26-Nov and 3-Dec			P-value^b^
	Average^a^	Variance	Std. error	Average^a^	Variance	Std. error	
**Proteobacteria**	52.8035	1.87	0.9681	84.7133	0.67	0.5804	0.000167
**Actinobacteria**	20.6281	19.53	3.1252	7.3514	11.41	2.3886	0.036333
**Firmicutes**	16.2664	0.66	0.576	2.924	0.78	0.6253	0.017222

^a^ Average proportion of the phylum during indicated date.

^b^ P<0.05 means that the proportion of indicated phylum has statistically difference.

### Characterization of bacterial community at the genus level

**Fig 4:**
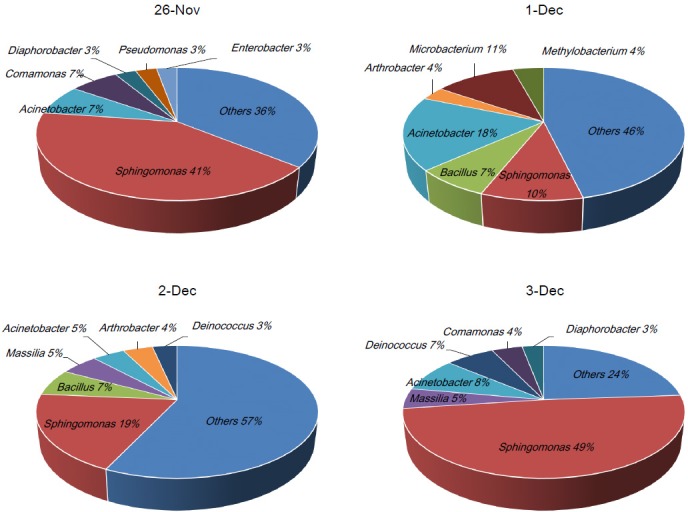
Bacterial communities (%) at the genus level in the Asian dust and non-Asian dust event samples. Other genera present lower than 2% of total bacteria community.

Next, we characterized the bacterial community at the genus level in the collected samples. For the analysis, we classified the pyrosequencing data at the genus level and confirmed that a total of 646 genera were present. Among them, 12 genera were found to make up more than 2% of the total proportion, as shown in Fig 4 and Table 3. During the non-Asian dust events, genus *Sphingomonas* belong to phylum Proteobacteria was predominantly present at the proportion of 45%, and genera *Acinetobacter*, *Comamonas*, *Deinococcus*, and *Diaphorobacter* were present at proportions of 7.8%, 5.4%, 3.3%, and 2.9%, respectively. Although the genus *Sphingomonas* was still present predominantly in the Asian dust event samples, the proportion in the total genera was largely decreased to 14.6%. Instead of the decrement, genera *Bacillus*, *Arthrobacter*, *Microbacterium*, and *Methylobacterium* were increased by 6.8, 4.8, 2.9, and 17.6 times, respectively, in the Asian dust event samples. To determine the significantly different genera in their proportions between the two groups, we performed Metastats analysis. As a result, genera *Bacillus* and *Arthrobacter* were found to be significantly increased by 6.8 and 4.8 times, respectively (Table 4). In addition, among various genera that have proportion of lower than 2%, genera *Blastocatella* (1.99% of the Asian dust event samples) and *Planomicrobium* (1.43% of the Asian dust event samples) were significantly increased by 16.2 and 6.9 times, respectively. These results reveal that genera *Bacillus* and *Planomicrobium* belonging to the phylum Firmicutes and *Arthrobacter* belonging to the phylum Actinobacteria were major parts of the bacterial community transported with dust particles in South Korea during the December 2014 Asian dust event. In particular, these genera have been known to find arid or semi-arid regions, suggesting that the feature of habitat may contribute a relatively high proportion of the genera in the Asian dust samples [35–37]. Among the identified genera, *Bacillus* has been reported to induce severe diseases such as anthrax and food poisoning caused by *Bacillus anthracis* and *Bacillus cereus*, respectively [38–41]. In our results, the most abundant *Bacillus* species is *Bacillus circulans* (2.6%, data not shown). Although there are a few cases which reveal *Bacillus circulans*-associated diseases, a fatal sepsis case in an immunocompromised patient is reported [42, 43]. A relationship between disease and the other genera identified in this result is not well understood, but several reports have showed that the genus *Arthrobacter* is found in clinical samples [44, 45]. An accumulation of bacterial information by continuous monitoring during Asian dust and non-Asian dust events should be necessary for establishing a relationship between human health and bacterial community during Asian dust events.

**Table 3:** Bacterial communities (%) at the genus level in the Asian dust and non-Asian dust event samples.

Genus	Bacterial communities (%)			
	26-Nov (non-Asian dust)	1-Dec (Asian dust)	2-Dec (Asian dust)	3-Dec (non-Asian dust)
***Sphingomonas***	41.44999	9.832842	19.3573	48.96853
***Bacillus***	Tr^a^	7.262256	6.541344	Tr
***Massilia***	Tr	Tr	5.182269	4.962867
***Acinetobacter***	7.373779	17.98005	4.458275	8.322527
***Arthrobacter***	Tr	3.286979	4.064524	Tr
***Deinococcus***	Tr	Tr	3.378636	6.695744
***Microbacterium***	Tr	11.1673	Tr	Tr
***Methylobacterium***	Tr	4.228122	Tr	Tr
***Comamonas***	6.699684	Tr	Tr	4.196629
***Diaphorobacter***	2.888981	Tr	Tr	2.970647
***Pseudomonas***	2.916495	Tr	Tr	Tr
***Enterobacter***	2.820195	Tr	Tr	Tr
**Others**	35.85087	46.24245	57.01766	23.88306

^a^ Tr, trace, indicates a proportion lower than 1%.

**Table 4:** Metastats analysis at the genus level in the Asian dust and non-Asian dust event samples.

Genus	1-Dec and 2-Dec (Asian dust)			26-Nov and 3-Dec			P-value^b^
	Average^a^	Variance	Std. error	Average^a^	Variance	Std. error	
***Bacillus***	6.9018	0.26	0.3605	1.0209	0.11	0.231	0.025584
***Arthrobacter***	3.6758	0.3	0.3888	0.7595	0.04	0.1347	0.039053
***Blastocatella***	1.9934	0.05	0.1532	0.1228	0.01	0.054	0.027008
***Planomicrobium***	1.4293	0.02	0.0949	0.2063	0.03	0.1238	0.036915

^a^ Average proportion of the genus during indicated date.

^b^ P<0.05 means that the proportion of indicated genus has statistically difference.

## Conclusions

In this study, we characterized the airborne bacterial community during Asian dust and non-Asian dust events from November to December 2014. As a result, we found that the diversity of the airborne bacterial environment was increased by newly transported dust particles, which contain various microorganisms, during the Asian dust event, leading to a decrement of the proportion of the predominant bacteria in the airborne environment. During the Asian dust event on December 2014, the proportion of predominantly presented phylum Proteobacteria was significantly reduced compared to the non-Asian dust event in November and December 2014. Meanwhile, phyla Actinobacteria and Firmicutes, especially genera *Bacillus*, *Arthrobacter*, and *Planomicrobium*, were newly introduced with dust particles. In addition, although the proportion was comparatively lower than the microorganisms mentioned above, various microorganisms were also transported during the Asian dust event. Indeed, the small proportion of newly transported microorganisms cannot be disregarded because the total amount (16S rRNA gene copy number) of microorganisms was dramatically increased (several hundreds to thousands) in the airborne environment during the Asian dust event [33]. In a further study, continuous monitoring of Asian dust and non-Asian dust event particles should be performed to determine the biological characterization of Asian dust particles. Moreover, various environmental effects, including soil environment, human health, and industrial problems, affected by the precipitated microorganisms should also be evaluated.

## Supporting Information

S1 File Raw sequence file for bacterial 16S rRNA gene sequences during November 26, December 1, December 2, and December 3, 2014, South Korea. Files named by Nov 26 and Dec 3 are non-Asian events, and files named by Dec 1 and Dec 2 are Asian dust events. The files were submitted into the INSDC SRA (BioProject and SRA accession numbers are PRJNA360153 and SRP096119, respectively).
(ZIP)
